# Psychiatric Care in NHS Lothian - Is It Working? an Evaluation of the Service

**DOI:** 10.1192/bjo.2026.11614

**Published:** 2026-06-30

**Authors:** Hania Afzal, Rachel Brolly, Aiste Gedvilaite, Kenneth Murphy, Douglas Murdie

**Affiliations:** 1 University of Edinburgh, Edinburgh, United Kingdom; 2 NHS Lothian, Edinburgh, United Kingdom

## Abstract

**Aims:**

Our aim was to evaluate the psychiatric service provided by NHS Lothian, specifically the Royal Edinburgh Hospital (REH), which provides inpatient care for patients in Edinburgh, East Lothian and Midlothian.

**Methods:**

Data was collected from NHS Lothian analytical services, the Director of Public Health Annual Report 2024, NHS Benchmarking Network findings (2022/23), the Scottish Government Mental Health Inpatient Census 2024, the National Records of Scotland and the World Health Organisation (WHO). Data was anonymised in line with NHS Information Governance Policy.

We evaluated the service provided for working age adults (18-64 years old) in REH using key performance indicators (KPIs) including bed occupancy, re-admission rates, length of stay (LOS) and staffing. Results were compared with other health boards in Scotland.

**Results:**

The relevant population is approximately 480000 people. Since 2001, East Lothian, Midlothian and Edinburgh are the fastest growing populations in Scotland (27.7%, 23.4% and 18.2%).

In 2014, European Union members had on average 77 beds per 100000 population. The United Kingdom had 46 beds per 100000 population. In 2015, the REH was rebuilt with a 20% reduction in capacity, with 105 acute adult beds. This equates to 22 beds per 100000 population (Scotland mean=28/100000).

The Royal College of Psychiatrists recommends a maximum safe bed occupancy of 85%. Mean bed occupancy for acute adult beds in REH has increased from 96.2% in 2021 to 109.5% in 2025. This was last under 100% in March 2022.

Over the same timescale, mean LOS has increased (19 days to 27 days) and mean admissions per month have fallen (154 to 131).

11% of patients required re-admission within 30 days of discharge (Scotland mean=8%). Lothian had the highest percentage of compulsory admissions at 33% (Scotland mean=19%).

Turnover of hospital staff was 18% (Scotland mean=15%). Community teams had 33 staff per 100000 population (Scotland mean=76/100000) with 20% vacancies (Scotland mean=17%).

**Conclusion:**

There has been a failure across all KPIs. Re-admissions, compulsory admissions and staffing are all worse than the Scottish average and bed occupancy has not been below 100% for 4 years.

Population growth puts pressure on all services, but NHS Lothian has failed to adjust for this to provide safe and effective inpatient care. Low community staffing only exacerbates the problem.

These findings indicate the need for review of managerial and funding decisions for psychiatric services in NHS Lothian to optimise patient care.

